# Self-Supporting

**Published:** 1892-02

**Authors:** 


### SELF-SUPPORTING.

It is pleasant and hopeful to note that so many young women are learning to value the mental powers and the education that will make it possible for them to support themselves if the necessity for doing so should arise. The daughters of comparatively wealthy men are not infrequently found assisting their fathers in the office or counting room as typewriters or accountants.

Many so called fashionable ladies make their own dresses and hats, we are told, having gone through a regular course of instruction in the art of millinery and dressmaking. An instance recently came to the notice of the writer that has in it a lesson for women who give no thought to the state of dependence to which they would be reduced if their parents or husbands should die, leaving them unprovided for.

A lady who had a beautiful home and three little children, and whose husband was supposed to be comparatively wealthy, one day found herself a widow and almost penniless, her husband having engaged in unfortunate speculations just before his death.

The lady's friends were profuse in their offers of sympathy, while wondering "what in the world she would do now."

She knew just what she would do. Within a month she had opened a millinery establishment that at once became very popular and profitable, for the bonnets she had worn in the past had been such models of elegance that her fashionable friends were glad to take advantage of her good taste. They never dreamed that she had made those bonnets herself, nor did they know that she had privately given herself a very good business education.—*Ex.*

